# Systems analysis and improvement to optimize pMTCT (SAIA): a cluster randomized trial

**DOI:** 10.1186/1748-5908-9-55

**Published:** 2014-05-08

**Authors:** Kenneth Sherr, Sarah Gimbel, Alison Rustagi, Ruth Nduati, Fatima Cuembelo, Carey Farquhar, Judith Wasserheit, Stephen Gloyd

**Affiliations:** 1 Department of Global Health, University of Washington Schools of Medicine and Public Health, 1705 NE Pacific St, Seattle, WA 98195, USA; 2 Health Alliance International (HAI), 1107 NE 45th St, Suite 350, Seattle, WA 98105, USA; 3 Network of AIDS Researchers of Eastern and Southern Africa (NARESA), Nairobi, Kenya; 4 Department of Paediatrics, University of Nairobi, Nairobi, Kenya; 5 Community Health Department, School of Medicine, Eduardo Mondlane University, Avenida Salvador Allende 702, Maputo, Mozambique; 6 Department of Medicine, University of Washington School of Medicine, 1959 NE Pacific Street, Seattle, WA 98195, USA; 7 Department of Epidemiology, University of Washington School of Public Health, 1959 NE Pacific Street, Seattle, WA 98195, USA

**Keywords:** Systems analysis, Quality improvement, pmtct, Value stream mapping, Cascade analysis, Cluster randomized trial, Industrial engineering, Implementation science, Mozambique, Kenya, Côte d’Ivoire

## Abstract

**Background:**

Despite significant increases in global health investment and the availability of low-cost, efficacious interventions to prevent mother-to-child HIV transmission (pMTCT) in low- and middle-income countries with high HIV burden, the translation of scientific advances into effective delivery strategies has been slow, uneven and incomplete. As a result, pediatric HIV infection remains largely uncontrolled. A five-step, facility-level systems analysis and improvement intervention (SAIA) was designed to maximize effectiveness of pMTCT service provision by improving understanding of inefficiencies (step one: cascade analysis), guiding identification and prioritization of low-cost workflow modifications (step two: value stream mapping), and iteratively testing and redesigning these modifications (steps three through five). This protocol describes the SAIA intervention and methods to evaluate the intervention’s impact on reducing drop-offs along the pMTCT cascade.

**Methods:**

This study employs a two-arm, longitudinal cluster randomized trial design. The unit of randomization is the health facility. A total of 90 facilities were identified in Côte d’Ivoire, Kenya and Mozambique (30 per country). A subset was randomly selected and assigned to intervention and comparison arms, stratified by country and service volume, resulting in 18 intervention and 18 comparison facilities across all three countries, with six intervention and six comparison facilities per country. The SAIA intervention will be implemented for six months in the 18 intervention facilities. Primary trial outcomes are designed to assess improvements in the pMTCT service cascade, and include the percentage of pregnant women being tested for HIV at the first antenatal care visit, the percentage of HIV-infected pregnant women receiving adequate prophylaxis or combination antiretroviral therapy in pregnancy, and the percentage of newborns exposed to HIV in pregnancy receiving an HIV diagnosis eight weeks postpartum. The Consolidated Framework for Implementation Research (CFIR) will guide collection and analysis of qualitative data on implementation process.

**Discussion:**

This study is a pragmatic trial that has the potential benefit of improving maternal and infant outcomes by reducing drop-offs along the pMTCT cascade. The SAIA intervention is designed to provide simple tools to guide decision-making for pMTCT program staff at the facility level, and to identify low cost, contextually appropriate pMTCT improvement strategies.

**Trial registration:**

ClinicalTrials.gov NCT02023658

## Background

Despite cost-effective, efficacious interventions to prevent pediatric HIV infection, as well as large investments to scale-up pMTCT services in countries with the highest burden of HIV, pediatric HIV infection remains largely uncontrolled [1]. Efforts to expand pMTCT have led to gains in the number of facilities with pMTCT services, reaching 78% of all clinics with ANC in Mozambique [2], 44% in Côte d’Ivoire [3], and 58% in Kenya [4]. Despite this expansion, gaps along the pMTCT cascade limit its effectiveness, with low coverage of HIV counseling and testing in the study countries (reaching between 47% to 72% of estimated HIV-infected women), low maternal access to ART prophylaxis and triple-therapy for eligible women (44% to 72% of estimated HIV-infected pregnant women), and limited infant access to ART prophylaxis (reaching 33% to 59% of infants born to identified HIV-infected women in the study countries). Infant feeding practices, low post-partum use of family planning, weak linkages with HIV care, and sub-optimal integration with other effective ANC services further impede pMTCT effectiveness. As a result, pediatric HIV infection continues to be common, with mother-to-child HIV transmission estimated to occur in 24% of children born to HIV-infected women in Kenya [4], 29% in Côte d’Ivoire [5], and 27% in Mozambique [6].

Enhancing the implementation of pMTCT interventions may lead to dramatic improvements in infant and maternal outcomes through reducing drop-offs along the pMTCT cascade. However, intervention studies to test novel implementation approaches for pMTCT have been limited in scope and methodology, and there are few published reports that describe large-scale, rigorously evaluated efforts to improve pMTCT programs in real-world settings. Operational studies to date have largely focused on identifying determinants of poor adherence to care, such as how delays in identification of infected infants are associated with poor access to comprehensive HIV care and late initiation of combination antiretroviral therapy (cART) [7], or how sociodemographic factors impede pMTCT program participation [8]. Health systems research addressing operational barriers to pMTCT uptake has been largely descriptive, though has highlighted the importance of health systems access and systems inefficiencies as factors that limit the effectiveness of pMTCT services [9]. Further, studies have identified data management challenges indicating that inconsistent data flow impede their use for decision-making and improvement efforts by pMTCT service management [10]. A further limitation of the published literature is that operational improvements have generally been carried out in small pilot programs [11,12], are mostly hospital based [13], and have short follow-up periods [14,15].

There is a growing recognition that the most critical priority for improving the effectiveness of pMTCT services is to increase the number of women successfully passing through the multiple, sequential steps in the pMTCT cascade [16], which argues for approaches that optimize pMTCT system delivery and related HIV care services in order to increase access to existing, efficacious interventions. Several novel techniques that have recently been applied to healthcare settings have the potential to improve health outcomes by identifying and reducing system inefficiencies and improving program effectiveness in complex, multi-step health services, such as the pMTCT cascade. These tools, such as value stream or process mapping, and continuous quality improvement, have been adapted from industrial and systems engineering to improve manufacturing, and have led to dramatic and rapid increases in program efficiency through simple, low cost, iterative adaptations in process design and service delivery [17,18]. A common element of systems analysis and improvement approaches is to describe and understand the existing system to identify problems and risks, and to generate solutions. Process mapping engages health managers and workers to describe the discrete, sequential steps in multi-step health service delivery strategies, which then serve as a basis for identifying contextually-appropriate systems innovations to improve system functioning [19].

A hallmark of systems analysis and improvement approaches is encouraging the participation of frontline health workers in analyzing their system, as well as defining, implementing, and evaluating improvement strategies using simple, locally accessible, and relevant data. Previous research, including research from resource limited settings, has found that engaging local health staff in health systems analysis and identification of adaptations for systems improvement leads to strategies that are more appropriate, effective, and sustainable than health systems analysis interventions that do not involve local staff [18]. By building a shared understanding of how work is really carried out, process mapping helps to build common organizational values and goals, which has been found, along with the involvement of senior management champions, to be associated with improved health service delivery and patient-level outcomes at the facility level [20,21].

To date, the majority of quality improvement methodologies based on the experiences from systems engineering have been implemented in healthcare systems in high income countries, and despite their widespread use, there is insufficient evidence that these techniques improve the quality of individual health provider care [22-25] and/or institutional performance [26-32]. Experience with these systems analysis and improvement techniques is limited but growing in resource-constrained settings [33,34], including in applications to pMTCT services [35,36]. Often, evidence on the effectiveness of these approaches is anecdotal or relies on descriptive studies that document changes in process measures in a small set of intervention facilities without a counterfactual, and have shown mixed results in improving utilization and quality of care measures [18,37-43]. Given the limited evidence on systems analysis and improvement interventions, despite their potential for optimizing complex, linked systems like pMTCT, this area merits further study [44].

### Goals and objectives

The goal of this study is to strengthen pMTCT programs in the three study countries by applying and evaluating systems analysis and improvement approaches, to build on existing experience with these techniques.

Our primary hypothesis is that identifying modifiable barriers to completing steps in the pMTCT cascade and applying locally-defined innovations will lead to measurable improvements in the performance of pMTCT services over and above those observed during the same time period in control facilities. The primary outcomes for this pragmatic trial focus on the proportion of women and children pairs successfully progressing through the pMTCT cascade from HIV screening at first antenatal care (ANC) visit, to successful receipt of antiretroviral medicines, and ending with HIV screening in infants. These measures are sensitive to the systems analysis and improvement (SAIA) intervention, readily measurable, and represent steps that are essential for successful prevention of HIV acquisition in children. Additional qualitative data collection embedded into the trial is designed to describe intervention characteristics that may influence the success of the SAIA intervention, the implementation environment, and the implementation process.

## Methods

The SAIA intervention entails mentored, iterative application of a systems analysis tool and related improvement approach to provide facility-level pMTCT staff and managers with a holistic view of their system’s performance, identify which steps in the pMTCT cascade are the highest priority for improvement and which bottlenecks are modifiable, and test contextually appropriate solutions. By mentoring facility staff to identify and test solutions, it is expected that this overall analysis and optimization process will lead to rapid and sustainable improvements in pMTCT service delivery, quantified using routinely available indicators. The findings coming from three diverse countries in sub-Saharan Africa, coupled with additional implementation process measures, will provide practical results that are directly applicable to pMTCT programs in diverse settings.

This intervention incorporates systems engineering techniques that provide a systems view combined with iterative improvement cycles, which have been designed to holistically capture complex real-world systems and improve their functioning. These methodologies are also intended to be user friendly and simple to apply in order to engage frontline health workers and facility managers in the process of identifying bottlenecks and solutions, and testing sustainable solutions that are within their scope of control.

The pragmatic study design and analytical approach represent an appropriate and robust attempt to evaluate the study intervention. A cluster randomized design is feasible and efficient, will adequately enable the detection of improvements in pMTCT performance associated with a facility-based intervention, and will reduce the risk of potential confounding related to assignment of health facilities to receive the intervention. Furthermore, the inclusion of three diverse countries in sub-Saharan Africa will improve the relevance and applicability of trial results in high need countries.

### Trial design

This study employs a two-arm, 1:1 cluster randomized trial design to assess the effectiveness of the SAIA intervention. The unit of intervention is the health facility. A total of 36 health facilities are part of the trial, including 18 intervention and 18 comparison facilities (six intervention and six comparison facilities per country).

### Study facilities and setting

**Figure 1:**
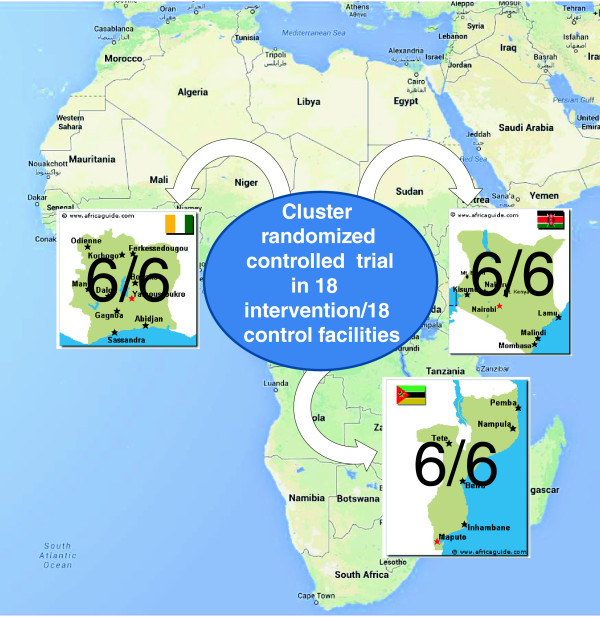
**Map of the Systems Analysis and Improvement Approach (SAIA) study countries.** Intervention and comparison facilities split equally across the three study countries (six intervention and six comparison facilities per country).

The SAIA intervention is applied at the facility-level. Through improved pMTCT services, it aims to improve outcomes for pregnant and postpartum mothers and their infants. Study facilities are split evenly by intervention and comparison group across three sub-Saharan African countries—Côte d’Ivoire, Kenya and Mozambique (Figure 1). These three countries were chosen to provide heterogeneous implementation settings in order to generate evidence on the SAIA intervention across multiple countries from diverse geographic regions, with different health sector designs, varying levels of resource investments, and different patterns of HIV burden.

### Côte d’Ivoire

Study facilities are located in three northern regions of Côte d’Ivoire (Gbêké, Hambôl, Poro-Tchologo-Bagoue) which had an estimated adult HIV prevalence of 4.4%, 4.4%, and 2.5% respectively in 2011 – 2012 [45]. PMTCT services in Côte d’Ivoire are provided primarily through public sector health facilities, as well as some private facilities. In 2011, 734 health facilities actively provided PMTCT services in Côte d’Ivoire, of which 113 are located in the study regions. The World Health Organization’s PMTCT Option B regimen was adopted as national policy in November 2012.

### Kenya

Study facilities were selected from Nairobi city and Coast province, which had an estimated adult HIV prevalence of 4.9% and 4.3%, respectively, in 2012 [46]. PMTCT services in Kenya are primarily provided through public sector and private not-for-private health facilities, and use a mixed approach that includes the World Health Organization’s Option B + strategy in high volume facilities, with Option B and A in lower volume facilities.

### Mozambique

Study facilities were selected from Dondo and Nhamatanda districts, and Beira City, located along the heavily populated Beira corridor in Sofala province, Mozambique. Sofala has an estimated average adult HIV prevalence of 15.5%, which is higher in densely populated, urban areas [47]. The Mozambique National Health Service has a broad network of health facilities, and is the principal provider of formal health services in Sofala, providing over 98% of outpatient services in the province [48]. Utilization of primary healthcare services, including maternal and child health services, is high, and an estimated 95% of women have at least one ANC visit, and 71% deliver in institutional settings [49]. Since the launch of multiple HIV prevention, care and treatment strategies between 2002 – 2004, HIV services (including pMTCT) have been rapidly scaled-up to achieve geographic coverage in an integrated fashion [50]. The Ministry of Health uses a mixed approach for pMTCT, including the World Health Organization’s Option B + in facilities with current cART capacity, and Option A in the remaining facilities.

### Randomization

All public and non-profit health facilities with pMTCT services in the study region in each country were considered for inclusion. Facilities were excluded if they were more than 20 kilometers from a main transport corridor to ensure frequent contact with the study team. Facilities were also excluded if an ongoing prospective study or similar systems analysis and enhancement technique were being implemented, and if facility managers and/or staff were unwilling to participate in the study. Consent for inclusion in the trial was sought from facility leadership after the randomization process.

**Figure 2:**
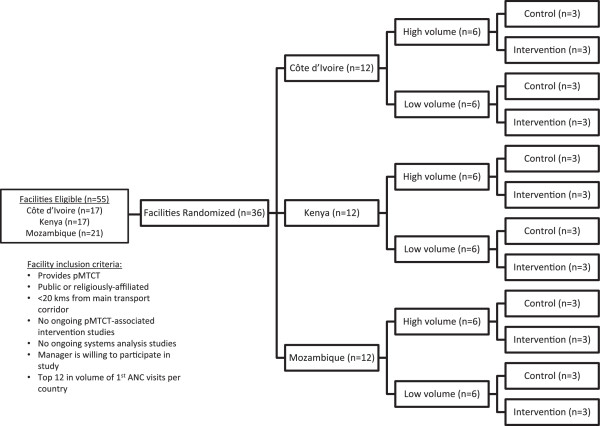
**Facility eligibility and randomization.** ANC: Antenatal care; KM: Kilometer; PMTCT: prevention of mother-to-child transmission (of HIV).

A total of 90 health facilities providing pMTCT (30 per country) provided the initial sample frame for this study (Figure 2). Study leadership from each country (including the project Principal Investigator) met in November 2013, in Beira, Mozambique, to define intervention and comparison groups. After removing facilities that did not meet the eligibility criteria, the remaining 55 facilities were ranked by country according to volume of first ANC visits. The top 12 facilities were selected in each country, and split into two groups of six facilities (upper and lower 50%, according to ANC volume). Three facilities from each group were assigned to receive the intervention and three to not receive the intervention in each country (using the random number generator in Excel®), totaling six intervention and six comparison facilities per country (18 intervention and 18 comparison facilities in total).

### Intervention description

**Figure 3:**
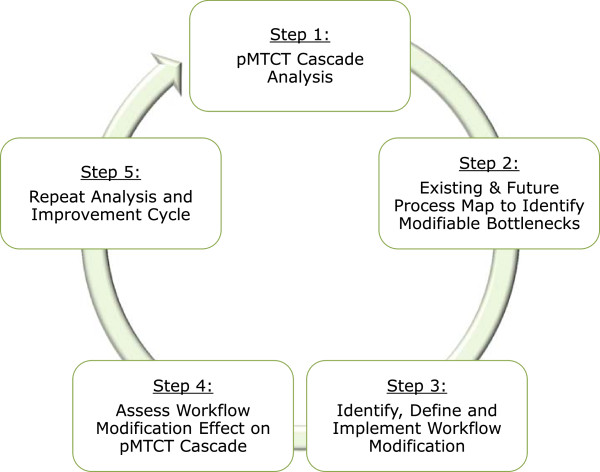
Five steps of the Systems Analysis and Improvement Approach (SAIA intervention.

The SAIA intervention is a facility-based, five-step, iterative process designed to guide pMTCT staff and facility-level managers in understanding and improving their pMTCT services. The intervention incorporates tools to enable a systems view of pMTCT performance from ANC through postpartum care for mother-infant pairs and to discern modifiable barriers on which to focus improvement efforts, followed by continuous quality improvement cycles to guide facility personnel in the identification, implementation, and rapid evaluation of appropriate facility-level solutions (Figure 3).

### Step one: PMTCT cascade analysis to understand pMTCT performance, identify and prioritize areas for improvement

**Figure 4:**
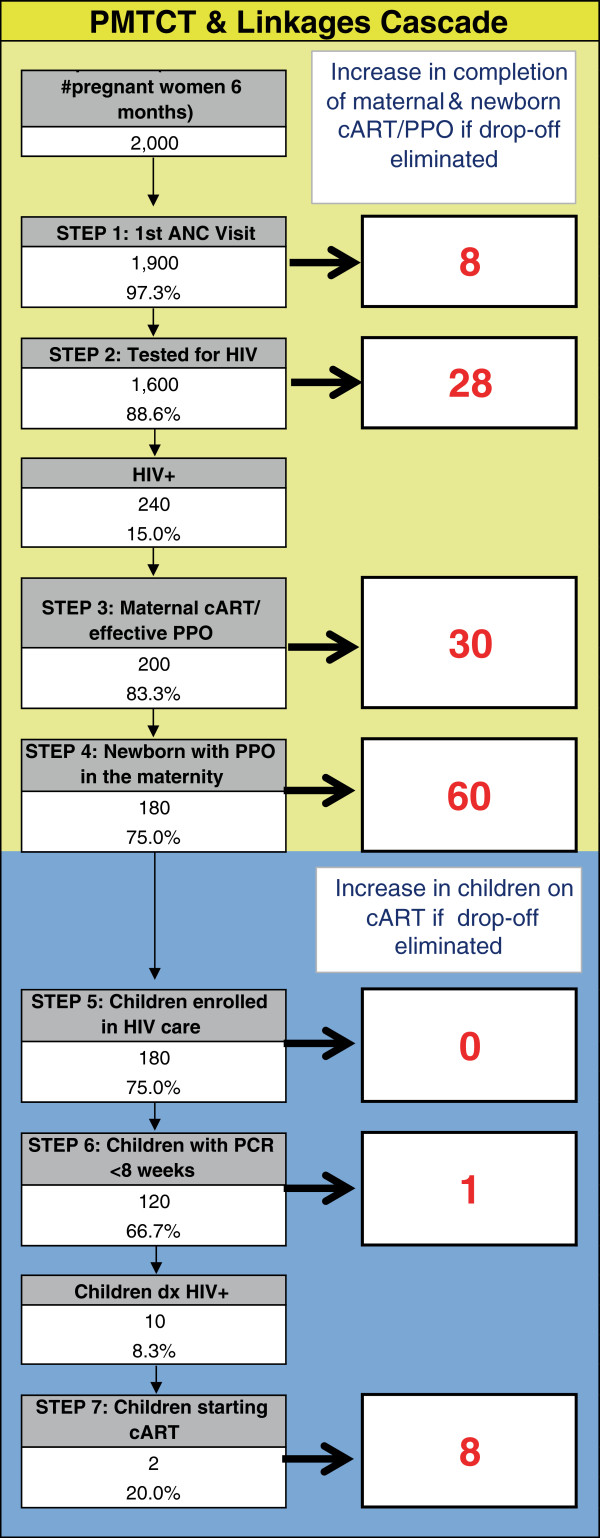
**PMTCT Cascade Analysis Tool (PCAT).** Demonstrates number lost and potential gains per step (if that step improved to 100%, holding the other steps constant) for the ANC➔maternity (yellow), and postpartum (blue) cascades. ANC: Antental care; cART: Combination anti-retroviral therapy; dx: Diagnosed; PPO: Prophylaxis.

An essential step for systems improvement is to engage facility-level pMTCT staff and managers in understanding their system’s performance and using performance data to identify and prioritize areas for improvement. A pMTCT cascade analysis tool (PCAT) was developed to provide this systems view and highlight priority areas to address within the pMTCT cascade, based on a similar approach representing the linkage between HIV testing and combination antiretroviral services in Mozambique (Figure 4) [51]. This Microsoft Excel^©^-based tool uses routinely collected facility data to calculate the number and proportion of women and children flowing through each step of the pMTCT cascade, broken down into the flow from ANC through birth, and the subsequent postpartum period. The PCAT is also automated to calculate the number lost at each step (difference between those eligible for and those successfully passing through each step), and to estimate the additional number of women and exposed infants who would complete all pMTCT steps if each step were individually improved; this calculation assumes that drop-offs for all other steps remain constant and rates of eligibility to pass to next steps are similar among those who do and do not pass through each step. As the first step of the SAIA intervention, the cascade analysis tool is used by study personnel to guide a discussion with facility-level staff to determine which steps in the pMTCT cascade are likely to have the largest effect in order to optimize overall pMTCT services.

### Step two: process mapping to identify modifiable bottlenecks at the facility level

**Figure 5:**
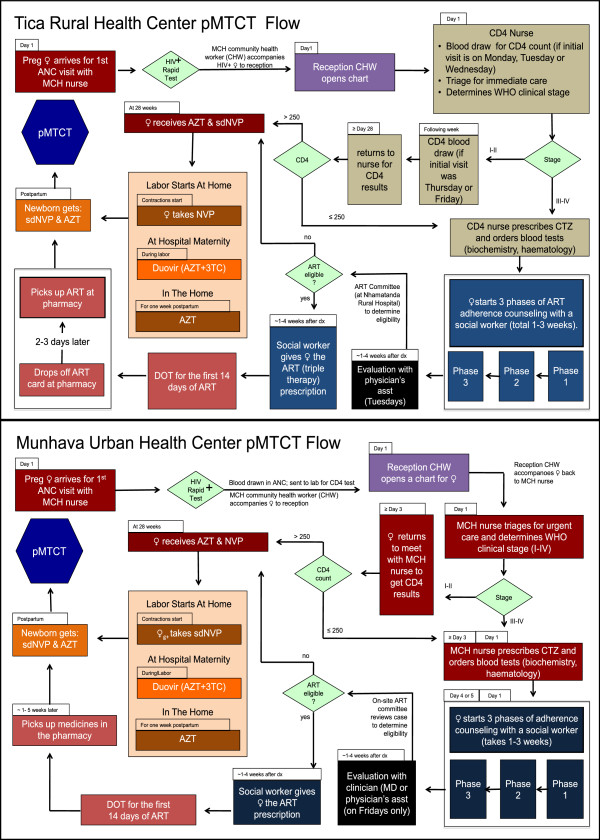
**Example of pMTCT process maps from two facilities in Sofala Mozambique.** Legend: Maps are from a medium-sized rural health center (Tica) and large urban health center (Munhava) in 2009, and demonstrate the flow of women from entry into antental care through receipt of antiretroviral prophylaxis or combination antiretroviral therapy.

To support facility-level staff in identifying specific bottlenecks in their pMTCT system, study teams will work with staff from the ANC, maternity, postpartum, and at-risk child care settings to map the existing flow of mother-infant pairs across these services (see Figure 5 for an example of two ANC flow maps from a large, urban and a smaller, rural facility in central Mozambique). By working with facility staff to explicitly describe the sequential, linked processes of care delivery at their facility, systems inefficiencies and potential solutions are highlighted, and health workers and managers are directly engaged in process improvement efforts. Each intervention facility’s existing pMTCT flow will be described on paper with input from multiple pMTCT staff, then be transferred to a computerized version using Microsoft Visio, and subsequently be discussed with pMTCT staff to confirm that it accurately represents the care processes in place and to identify inefficiencies that can be modified within each step.

### Step three: identify, define, and implement facility-specific workflow adaptations to eliminate modifiable bottlenecks

The final three steps of the SAIA intervention build off of continuous quality improvement methodologies. In step three, the PCAT analysis and process maps will be a focal point for brainstorming solutions with facility-level staff, mentored by study assistants trained in the SAIA intervention (training includes both didactic introduction to the intervention, and practical application of the tools during a one-week pilot phase in each country). It is expected that these solutions will lead to more efficient progression across the pMTCT cascade through process flow adaptation and simplification, task restructuring, service integration, and/or job aid introduction. Workflow adaptations will be selected based on their potential to lead to rapid and sustainable improvements in the targeted step in the pMTCT cascade, and feasibility of implementation, including being within the sphere of influence of facility management and pMTCT staff (Table 1). An implementation plan for the innovation will be described in writing by facility and study personnel, including a future state process map that reflects processes after the modification, to ensure consensus among facility staff on the contents of the solution, as well as clarity of operational design and roles and responsibilities among facility personnel. After identifying and defining the adaptation to be implemented, facility staff will implement the proposed changes.

**Table 1:** Illustrative facility-level workflow modifications

**Intended effect**	**Workflow modification**
Increase the proportion of women in ANC who receive HIV testing and counseling	Initiate a grouped HIV counseling protocol for pregnant women in the ANC service waiting area rather than providing individual counseling for all women within ANC services.
Increase the proportion of HIV-infected pregnant women with access to CD4 testing	Initiate CD4 blood draw for HIV-infected women by ANC nurses at the time of HIV diagnosis rather than referral to separate HIV clinics for enrolment and subsequent blood draw.
Increase the proportion of eligible HIV-infected pregnant women who initiate cART during pregnancy	Initiate cART for eligible pregnant women after the first counseling visit post determination of eligibility rather than requiring completion of three counseling visits before initiating cART (while maintaining subsequent counseling visits post-cART initiation).
Increase the proportion of HIV-exposed infants receiving HIV screening with PCR at six weeks of age	Attach ANC cards with HIV exposure data to the well child monitoring card at birth for institutional deliveries in order to systematically identify exposed infants at the six week well-child care visit.

ANC: antenatal care; cART: Combination anti-retroviral therapy; CD4: Cluster of differentiation 4; HIV: Human immunodeficiency virus; PCR: Polymerase chain reaction.

### Step four: assess workflow modification effect on pMTCT cascade

Using routinely reported pMTCT cascade data from the pMTCT step that was selected for improvement, facility staff will monitor improvements in relevant indicators for two to six weeks following the introduction of the facility-specific workflow adaptation. Monitoring data will be analyzed visually and using descriptive statistics after a short time period, relying on improvements in the absolute increase in the proportion of women or infants successfully progressing through the step of interest to capture large, rapid improvements associated with the introduction of the adaptation.

### Step five: repeat analysis and improvement cycles

Systems engineering process improvements are by definition iterative, entailing ongoing testing of innovations that respond to contextually specific, modifiable barriers. Facility staff will repeat steps one through five at the end of each change cycle, focusing on identifying new approaches to modify previously identified barriers, or if the first cycle was successful, focusing on improving priority bottlenecks identified in a repeated systems analysis.

### Adaptation of the analysis tool and improvement intervention

To ensure intervention adequacy and the implementation process, the SAIA intervention was piloted in five facilities for six months in Sofala, Mozambique. Through the piloting phase, the PCAT was adapted and translated from Portuguese into French and English, a toolkit was developed to support introduction of the intervention, and standard operating procedures were written to guide the implementation process and data collection procedures for the trial.

### Process for introducing the SAIA intervention

The SAIA intervention is designed to be introduced to each study facility over a four-day period (Table 2). On day one, trained study nurses hold a meeting with representatives from sectors of the health facility relevant to pMTCT, which may include ANC, maternity, postpartum care, at-risk child care, laboratory, and pharmacy staff. The specific composition depends on the individual health facility structure. At this initial meeting the study objectives and core components of the intervention are introduced, a calendar of activities for the week and across the six-month intervention period are developed, and PCAT results populated with the facility’s data from the most recent six months shared. On days two and three, study nurses work with facility staff to develop process maps for relevant services (ANC, maternity, postpartum care, and at-risk child care) with up to two health workers from each sector (including the sector in-charge and a second staff member who is knowledgeable about the health facility and are willing to participate in the exercise). A feedback meeting is held at the end of day three to have staff from each sector share their process maps, prioritize which sector to target with the initial improvement cycle, and brainstorm potential solutions. On day four, study nurses work with facility staff to prioritize solutions, and develop an implementation plan that explicitly records: the change(s) to be tested; the individual(s) responsible for each change; metrics to assess service improvement; a future process map to reflect the innovation; and a follow-up visit schedule. Subsequent follow-up visits are planned weekly for the first four weeks, bi-weekly for the next eight weeks, and subsequently according to the schedule agreed upon by facility staff and based on support needs.

**Table 2:** SAIA introduction schedule

**Activity**	**Day 1**	**Day 1**	**Day 2**	**Day 2**	**Day 3**	**Day 4**
	**AM**	**PM**	**AM**	**PM**		
Intro to SAIA and PCAT	X					
Process mapping						
ANC		X				
Maternity			X			
Postpartum/At-risk care				X		
Feedback session					X	
Implementation start						X

ANC: Antenatal care; HIV: Human immunodeficiency virus; PCAT: PMTCT cascade analysis tool; SAIA: Systems Analysis and Improvement Approach.

It is expected that each change cycle will require four to six weeks of testing to allow for sufficient time to detect desired effects on the pMTCT cascade, but to be short enough to allow for multiple iterations during the six-month intervention period. After each cycle, study staff will work with facility staff to review the original implementation plan, describe what actually occurred (including review of planned metrics to assess improvements), discuss what worked well and why, and identify what could be improved and how. The five-step SAIA intervention cycle will again be repeated to review PCAT data, update process maps, and develop the next change cycle. It is expected that between four and six change cycles will be implemented in each facility over the six-month intervention period.

### Study timeline

The total data collection and implementation period is designed to cover 21 months, which includes 12 months of pre-intervention data collection, six months of intervention implementation, and three months of post-intervention data collection. The 12-month timeframe for pre-intervention data collection was chosen to generate stable baseline study outcomes despite monthly temporal fluctuations in these measures. A total of six months was selected for implementing the study intervention to provide sufficient time for the intervention to be adopted and lead to improvements, to allow for a sufficient number of iterative system analysis and improvement cycles to lead to sustainable performance improvements (assuming that each cycle will last on average four to six weeks, this timeframe will allow for between four and six cycles per facility), and to be feasible to complete within the specified funding timeline.

### Study measures

#### Primary study outcomes

The three study outcomes include the proportion of women screened for HIV during their first ANC visit; the proportion of HIV infected women receiving antiretroviral medications during pregnancy (either bi-prophylaxis using AZT or combination antiretroviral therapy); and the proportion of infants born to HIV-infected women screened for HIV at six weeks postpartum (or two months in the case of Côte d’Ivoire, following national norms) (Table 3). These outcome measures were selected because they reflect successful progression through steps in the pMTCT cascade, they are likely sensitive to system-level improvements, and they represent steps that, if changed, would meaningfully alter patterns of HIV transmission or access to maternal and infant HIV care. Furthermore, these measures reflect national pMTCT protocols, and are therefore well understood and available in all three study countries. Data will be collected over 12 months before the intervention, during the six-month intervention, and three months after the intervention in both intervention and comparison facilities (21 time points and 36 measurements per time point).

**Table 3:** pMTCT outcome measures

**Study measure**	**Numerator/Denominator**
1. Uptake of HIV counseling and testing	$\frac{\#\mathrm{women}\;\mathrm{counseled}\;\mathrm{and}\;\mathrm{tested}\;\mathrm{for}\;\mathrm{HIV}\;\mathrm{in}\;\mathrm{their}\;\mathrm{first}\;\mathrm{ANC}\;\mathrm{visit}}{\#\mathrm{first}\;\mathrm{ANC}\;\mathrm{visits}}$
2. Use of appropriate ARVs in pregnancy for prophylaxis or initiation of cART	$\frac{\#\mathrm{HIV}‒\mathrm{infected}\;\mathrm{pregnant}\;\mathrm{women}\;\mathrm{starting}\;\mathrm{AZT}\;\mathrm{prophylaxis}\;\mathrm{or}\;\mathrm{cART}}{\#\mathrm{women}\;\mathrm{testing}\;\mathrm{HIV}‒\mathrm{positive}\;\mathrm{in}\;\mathrm{ANC}\;3\;\mathrm{months}\;\mathrm{previously}}$
3. Infant HIV determination	$\frac{\#\mathrm{infants}<6\;\mathrm{weeks}\;\mathrm{of}\;\mathrm{age}\;\mathrm{receiving}\;a\;\mathrm{PCR}\;\mathrm{test}}{\#\mathrm{women}\;\mathrm{testing}\;\mathrm{HIV}‒\mathrm{positive}\;\mathrm{in}\;\mathrm{ANC}\;5\;\mathrm{months}\;\mathrm{previously}}$

ANC: Antenatal care; ARV: Antiretroviral; AZT: Azidothymidine; cART: combination antiretroviral therapy; HIV: Human immunodeficiency virus; PCR: Polymerase chain reaction.

All primary outcome measures are calculated as a monthly average (continuous percentage between 0% and 100%). Notably, outcome measures for study measures on use of appropriate ARVs in pregnancy for prophylaxis or initiation of cART, and infant HIV determination, include numerators and denominators from different time periods due to time progression between service contacts that can be represented by available data. For the use of appropriate ARVs in pregnancy, the delay between the service contact in the numerator (the number of pregnant women receiving AZT or cART) and denominator (number of women testing HIV positive in ANC) is estimated to be two months. This two-month delay reflects a mean gestational age of first ANC visit of 25 weeks, or approximately three months before initiation of AZT or cART. The recommended timeframe for PCR testing of infants is on average five months after the first ANC visit, where the majority of pregnant women undergo HIV testing.

### Covariates

Facility-level covariates include those that were found to be associated with pMTCT performance in analyses that are part of the wider research project that includes the SAIA intervention trial, including patient volume, staffing levels, availability of laboratory tests, geographic location (urban or rural), availability of community-based support groups, year of pMTCT initiation, and pMTCT approach (Option B+/B/A).

### Implementation process measures

Further qualitative data will be collected during the implementation process, and at the end of the trial, to facilitate the interpretation of the primary study results and increase the applicability of the SAIA intervention to other settings. The qualitative implementation process measures were designed based on the domains of the Consolidated Framework for Implementation Research (CFIR) [52], and intended to provide contextual information on factors that are important for the intervention’s acceptability, penetration and adoption by pMTCT staff and facility leadership, as well as factors impacting the quality, feasibility, and sustainability of the intervention’s application. Descriptive information will be collected on how the SAIA intervention was introduced, facility-level innovations that were proposed for testing, what was actually implemented, what went well and why, and what could be improved. Likert scale measures (scaled between 1 to 10) as well as open-ended questions will focus on manager and staff impressions on the acceptability of the intervention, utility of the intervention, leadership support, involvement of other agencies, intervention timing, staff capability to implement the intervention, clarity of roles to implement the intervention, ability of the intervention to meet staff and patient needs, and what the intervention fails to address.

### Data sources

#### Primary study outcomes

Health facility registries from ANC, maternities, and postpartum child care services are the source for monthly performance measures pre-, during and post-intervention at the 36 study facilities. Data extraction from registries is performed by two trained study team members in each facility, and compared for consistency. In cases of inconsistency, data collection procedures are redone, until monthly totals are in agreement. All project data are double-entered into a Microsoft Access^©^ database by each in-country study team and sent to the Seattle-based support staff for warehousing. Additional quality control procedures are carried out on a monthly basis on the centralized database to identify cases of missing data or outliers.

### Covariates

Additional facility-level descriptive factors will be collected via interviews with facility managers and direct observation by trained study personnel using a data collection instrument designed for the purposes of this study. Covariates cover information on the facility (size, geographic location, public/non-profit managed), staffing levels and structure, service utilization, available auxiliary services (laboratory, pharmacy, community outreach), and other similar factors that may affect both the adoption and effects of the intervention.

### Implementation process

In addition to the routinely reported administrative data, in-country study teams will collect descriptive implementation process data on an ongoing basis using data collection instruments designed for the purposes of this study. A 14-item questionnaire using Likert scales (ranging from 1 to 10) and open-ended questions was developed to gather data relevant to the CFIR domains, and will be applied weekly for the first four weeks of the SAIA intervention, followed by monthly for the remaining five months, with facility managers and a rotating group of facility nurses. This questionnaire will be complemented by field diaries maintained daily by study teams, and post-intervention key informant interviews with study assistants and in-country study managers.

### Analysis

The primary analysis approach to assess the impact of the SAIA intervention will rely on paired t-tests comparing the average outcome measures over the 12 months preceding the intervention, compared with the average over the three months directly following the intervention period, for the three study outcomes. As a secondary analysis approach, segmented regression analysis that controls for baseline outcome levels and trend to compare the monthly averages before versus after the intervention will be carried out for all three study outcomes. Change points will include a node at the beginning of the intervention period to reflect the introduction of the intervention; two additional terms will be added to the model at the end of the six-month intervention period to indicate the level change and trend change post intervention. To account for a three-month lag in the impact of the intervention, data from the first three months post initiation of the intervention will be excluded from analysis. Sub-analyses will be carried stratified by country and facility size to assess subgroup effects.

Because facilities will be randomly selected and allocated to intervention and control facilities, we do not expect to perform adjusted analyses. However, descriptive characteristics will be compared between intervention and comparison facilities using student’s *t*-test and chi-square tests to assess potential imbalance between study arms, and subsequent analyses will consider covariates significant in bivariate analysis for inclusion in the final model in a stepwise fashion. In addition, because outcomes are reported as a monthly summary measure at the facility level, no additional procedures will be implemented to control for potential intra-class correlation at the cluster (health facility) level. Autocorrelation will be assessed using the Durbin-Watson statistic, and adjusted for if appropriate.

Descriptive analyses will include visual inspection of data for time trends across the study facilities, as well as within each facility, to identify trends, assess whether specific workflow adaptations led to improvements in the pMTCT cascade, and describe the sustainability of effects associated with individual adaptations. Those adaptations that are found to have dramatic or sustained effects will be documented as part of identifying best practices for pMTCT improvement. Exploratory bivariate analyses using student’s t-tests and chi-square tests will be carried out among facilities that showed large improvements in study outcomes during the six-month implementation period to identify descriptive factors that may be associated with adoption or may mediate the impact of the SAIA intervention.

Analysis of the implementation process data will include aggregate reporting of individual items from the Likert scales, as well as disaggregated analysis by study country, and by facilities found to have relatively better success with the intervention. The constant comparison method will be used to describe responses to qualitative data from open-ended questions, field diaries, and key informant interviews.

### Sample size

Sample size estimates are based on the health facility as the unit of analysis, and were calculated to detect the change in the proportion of HIV-infected pregnant women receiving antiretroviral medicines in pregnancy (arguably the most important endpoint of the study). Using program data from 2009 to 2010, we estimated a mean pre-intervention baseline of 50%, and predicted a mean post-intervention level of 70% for intervention facilities (mean change score = 20%) and 50% for control facilities (mean change score = 0%), and a standard deviation of yearly change scores of 11%. Assuming α = 0.05 and β = 0.80, the sample size is sufficient to detect an 11% and a 20% difference in mean change scores across the three countries and at the country level, respectively (Table 4).

**Table 4:** Sample size and detectable change in study outcomes

**Sample size (Intervention: Control)**	**Detectable alternative in gain scores across intervention and control facilities**
6:6	19.7%
12:12	13.2%
18:18	10.6%

### Ethics

This study was approved by the institutional review boards of the Ministry of Health of Côte d’Ivoire, Kenyatta National Hospital, and the Ministry of Health of Mozambique, and was determined to qualify for exempt status by the human subjects division at the University of Washington. The study was registered with ClinicalTrials.gov (NCT02023658).

### Trial status

Implementation of the SAIA intervention began in January 2014 in all study countries, and is being sequentially introduced in the 18 intervention facilities (six in each study country). At the time of submission, the intervention has been introduced in nine facilities (four in Côte d’Ivoire, two in Kenya, and three in Mozambique).

## Discussion

The SAIA study is designed as a pragmatic trial, testing an intervention to maximize the benefits of an efficacious intervention (pMTCT) by improving its implementation in real world settings. Though systems engineering techniques have been increasingly applied to health service delivery, there is limited comparative analysis using scientifically rigorous, prospective evaluation techniques to support their continued use. With its rigorous design across three sub-Saharan African countries, this randomized trial is uniquely positioned to further our understanding of the application of these innovative improvement techniques for widespread use in improving health delivery in resource-limited settings. The collection of implementation process measures guided by a standardized, accepted framework will provide valuable information to guide further application and potential scale-up of the SAIA intervention. Furthermore, by documenting facility-level innovations tested through the SAIA intervention, the trial also intends to identify potential strategies for further testing and wider implementation.

The SAIA intervention has a number of potential advantages for pMTCT services. As a flexible, user-friendly, and low-cost approach to systems analysis and improvement, the intervention can be applied to multiple contexts and changing ARV prophylaxis or treatment guidelines. The iterative nature of the intervention provides a framework that can lead to sustainable, long-term service improvements. In addition, because systems engineering techniques have the potential to improve services and strengthen linkages between services, the application of a stepwise analysis and improvement approach is especially appropriate for pMTCT services that require both progression through multiple sequential steps, and successful linkage with non-ANC services for continued HIV care.

The rollout of the SAIA intervention to date has generated great interest and willingness to adopt the intervention, with only one intervention facility refusing to participate in the trial. However, there have been notable practical challenges thus far, including the high level of existing research underway in the study areas, which reduced the number of facilities eligible for participation in the trial. Elections, conflict, floods, and end-of-year holidays delayed the introduction of the intervention in study countries. Furthermore, the initial plan to rely on routine monthly health facility reports as the primary data source for study outcomes met with resistance due to concerns with data quality. As a result, study outcomes are being directly sourced from health facility registries. An additional operational challenge has been the high burden already placed on staff at participating health facilities, which constrains both available time to participate in the preparation and SAIA implementation, as well as enthusiasm to take on additional process improvement efforts. Finally, pMTCT guidelines have shifted rapidly since the initial study protocol was developed to include the initiation of cART for all women with HIV in pregnancy independent of CD4 levels (Option B/B+). Therefore, the relative importance of CD4 testing changed. Consequently, CD4 testing became less relevant as a step in the PCAT and was dropped as a primary study outcome.

Despite the challenges, the results of the SAIA intervention trial are likely to be of substantial interest to policymakers, managers and partners working to improve the already rolled out pMTCT services. Furthermore, the intervention approach may serve as a model for other services that, like HIV, require successful linkages across multiple services, such as chronic non-communicable diseases that are of growing importance, yet under-addressed in low and middle-income countries.

## Abbreviations

ANC: Antenatal Care; ART: Combination Anti-Retroviral Therapy; AZT: Azidothymidine (Zidovudine; antiretroviral medicine); CD4: Cluster of Differentiation 4 (laboratory test); CFIR: Consolidated Framework for Implementation Research; DX: Diagnosis; HIV: Human Immunodeficiency Virus; PCAT: PMTCT Cascade Analysis Tool; PCR: Polymerase Chain Reaction (laboratory test); PMTCT: Prevention of Mother-to-Child HIV Transmission; PPO: Prophylaxis; SAIA: Systems Analysis and Improvement Approach.

## Competing interests

The authors declare that they have no competing interests.

## Authors’ contributions

KS, SGi, CF, JW, and SGl conceived of the study. AR and BW advised the analytic approach. All authors contributed to refining the study design and finalizing the protocol. KS drafted the final version of the paper. All authors read and authorized the final version.
